# Exploring the short-term influence of a proprietary oil extract of black cumin (*Nigella sativa*) on non-restorative sleep: a randomized, double-blinded, placebo-controlled actigraphy study

**DOI:** 10.3389/fnut.2023.1200118

**Published:** 2024-01-15

**Authors:** M. E. Mohan, Mohind C. Mohan, Prathibha Prabhakaran, S. Syam Das, I. M. Krishnakumar, P. S. Baby Chakrapani

**Affiliations:** ^1^Department of General Medicine, BGS Global Institute of Medical Sciences, Kengeri, India; ^2^Centre for Neuroscience, Cochin University of Science and Technology, Cochin, Kerala, India; ^3^Department of Biotechnology, Cochin University of Science and Technology, Cochin, Kerala, India; ^4^R&D Centre, Akay Natural Ingredients, Cochin, Kerala, India; ^5^Centre of Excellence in Neurodegeneration and Brain Health, Cochin, Kerala, India

**Keywords:** actigraphy, black cumin, insomnia, non-restorative sleep, non-refreshing sleep, *Nigella sativa*, thymoquinone

## Abstract

**Background:**

*Nigella sativa* (black cumin, or black seed) is popularly known as the seed of blessings in the Arab system of medicine. Though not widely recommended for sleep, a unique proprietary black cumin extract (BlaQmax®/ThymoDream™; BCO-5) has been shown to be helpful in the management of stress and sleep issues.

**Methods:**

This randomized, double-blind, placebo-controlled trial aimed to investigate the efficacy of BCO-5 on the sleep quality of volunteers characterized with a self-reported non-restorative sleep disorder. Healthy male and female participants (*n* = 70), aged 18-65 years (BMI 22-28 Kg/m^2^) were randomized to either placebo or BCO-5 (*n* = 35/group). Both interventions were supplemented at 200 mg/day for seven days. Actigraphy and a validated restorative sleep questionnaire (RSQ-W) were used to monitor the influence of BCO-5 on sleep.

**Results:**

Compared to placebo, BCO-5 significantly improved sleep quality, as evidenced by both intra-group and inter-group analyses of the actigraphy data. The relative improvements observed were sleep efficiency (7.8%, *p* < 0.001), total sleep time (19.1%, *p* < 0.001), sleep onset latency (35.4%; *p* < 0.001), and wake-after-sleep-onset (22.5%; *p* < 0.001) compared with placebo. BCO-5 also improved sleep by 75.3% compared to baseline (*p* < 0.001) and by 68.9% compared to placebo (*p* < 0.001), when monitored by RSQ-W. BCO-5 was well-tolerated with no reports of side effects or toxicity.

**Conclusion:**

BCO-5 significantly improved non-restorative sleep in seven days, indicating its potential role as a natural sleep aid.

## 1 Introduction

Sleep is universally perceived to be an essential health factor. Inadequate sleep is associated with multiple health issues including obesity, type II diabetes, metabolic syndrome, depression, cardiovascular issues, and impaired cognitive function (1–3). Conditions such as stress, depression, and anxiety activate hypothalamic–pituitary–adrenal (HPA) axis and produces cortisol, which is considered as an important factor in the sleep deprivation (4–6). Sleep deprivation can lead to weight gain or obesity through elevated ghrelin and decreased leptin secretions (7). Various studies have demonstrated that progressively longer (≥ 9 h/night) or shorter (≤ 5 h/night) sleep spans are associated with age-specific mortalities (8, 9). In the pre-COVID period, nearly 70 million adults in the US and 45 million in Europe were estimated to suffer from sleeplessness (10, 11). The outbreak of the COVID-19 pandemic has severely affected sleep quality (12). Sleep disturbances pose serious risks to public safety in terms of, lack of concentration, absenteeism, loss of productivity and accidents. Extensive internet usage, late bedtimes, daytime sleep, sedentary lifestyle, and stress have been identified as the major factors affecting sleep (13). Nonrestorative sleep (NRS) is a common sleep disorder which refers to the subjective feeling that sleep has not been adequately rejuvenating or restful. It is typically considered as a secondary manifestation of insomnia according to international classification of sleep disorders (14, 15). Nonrestorative sleep has consistently been recognized as a distinct component of insomnia in the Diagnostic and Statistical Manual of Mental Disorders (DSM-4) (16, 17). Nonrestorative sleep arises from disrupted sleep and is indicative of the human body’s inability to recover from both physical and mental fatigue. Individuals experiencing nonrestorative sleep are more susceptible to impairments in daily activities, decline in cognitive functions, daytime sleepiness, mood swings, and fatigue (15). Consequently, nonrestorative sleep is considered to have a detrimental impact on overall quality of life. Modern treatment approach aims at improving/restoring the sleep.

Currently approved over-the-counter sleep therapy drugs include benzodiazepine receptor agonists and non-benzodiazepines (18). However, these drugs have been reported to be associated with various side effects, including drowsiness, nausea, dizziness, nightmares, agitation, headache, somnolence, fatigue, and dependency (19–21). Long-term exposure to these drugs is associated with dependency, nocturnal confusion, daytime dysfunction, and rebound insomnia (RIS) (22–24). Herbal remedies have garnered considerable interest in this context. *Nigella sativa* (black cumin/black seed/kalonji) is a widely distributed medicinal and culinary spice found in North Africa, Middle East, Europe, and Asia. Black cumin seeds, powder, paste and oils have been traditionally used since ancient times for the treatment of various diseases such as cardiovascular, respiratory, rheumatism, headache, back pain, anorexia, amenorrhea, mental illness, eczema and hypertension. A number of phytochemicals including thymoquinone, thymohydroquinone, thymol, carvacrol, *p*-cymene, nigellidine, saponins, and flavonoids have also been identified in black cumin (25, 26). Black cumin oil and its major bioactive component, thymoquinone, have been found to possess anti-inflammatory, anti-arthritic, anti-allergic, anti-microbial, anti-diabetic, hypolipidemic, hepatoprotective, neuroprotective, and anticancer properties (27–29). Recently, we showed that a unique black cumin extract composition (BCO-5, registered as BlaQmax®/ThymoDream™) acts as a calming agent and helps manage sleep disorders in human volunteers when consumed at a dose of 200 mg/day (30). It was also shown to be safe when a randomized placebo-controlled study was performed for three months at a dose of 200 mg/day (31). Acute and sub chronic toxicity studies have indicated its safety for human consumption at 900 mg/day per adult (32). Thus, the present randomized, double-blind, placebo-controlled trial was conducted on healthy volunteers with a non-restorative sleep pattern to investigate the influence of BCO-5 on sleep parameters, sleep quality, and sleep efficiency. The study used 200 mg/day dosage for just seven days and employed actigraphy, a non-invasive screening tool worn like a wristwatch, to monitor the effect.

## 2 Materials and methods

### 2.1 Study material

Dried black cumin seeds collected from selected farms in India, where they were cultivated following good agricultural practices, were authenticated by a pharmacognosist and a batch sample was deposited at the herbarium of Akay Natural Ingredients, Cochin, India (AK-NS-018). Authentication was performed using high-performance thin-layer chromatography densitometric analysis (CAMAG HPTLC system, Switzerland) in comparison with a standard reference material, as previously reported (31). The black cumin extract (BCO-5) was manufactured in a Good Manufacturing Practices (GMP)-certified plant in India (Batch no: BCOQ 32/21 dated 12/04/2021) and was provided by Akay Natural Ingredients, Cochin, India.

High-performance liquid chromatography (HPLC; Shimadzu Analytical India Private Limited, Mumbai, India) fitted with a reverse phase column (250 × 4.6 mm, 3 μm, Phenomenex, Hyderabad, India) and photodiode array (PDA) detector was used to measure thymoquinone (TQ) content. Other key bioactive terpenes and terpenoids were identified, confirmed, and quantified using gas chromatography-tandem mass spectrometry (GC–MS/MS) (Shimadzu Analytical India Private Limited, Mumbai, India) (30). All solvents used for the analysis were of HPLC grade and were procured from M/s (Sigma-Aldrich, Bangalore, India). Analytical standard of thymoquinone (CAS No:490-91-5) was purchased from Sigma-Aldrich (Bangalore, India).

### 2.2 Study design and protocol

**Figure 1:**
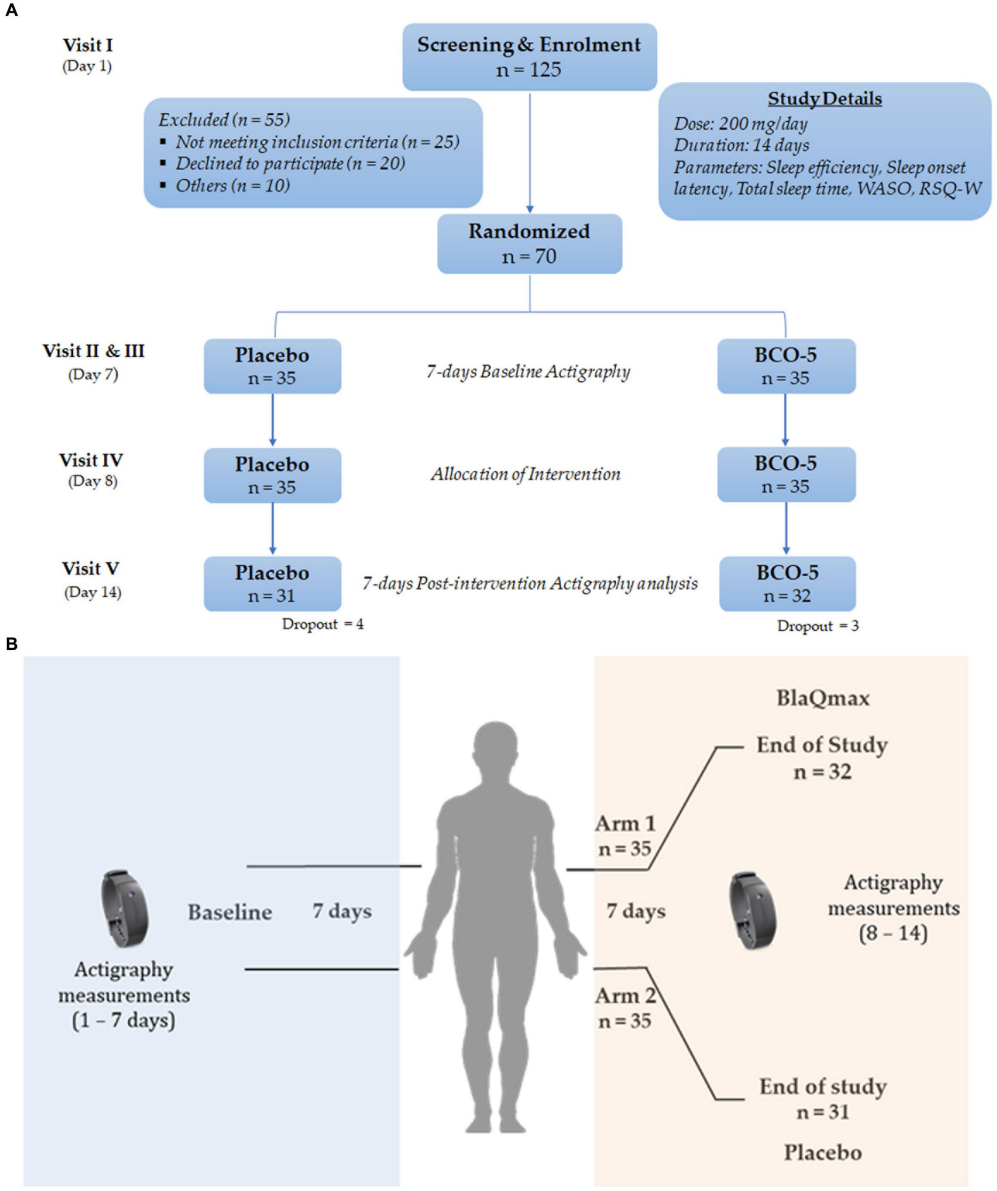
**(A)** Consort flow diagram depicting study design. **(B)** Schematic representation of cohort study design.

Identical soft gelatin capsules containing either BCO-5 or placebo (200 mg/capsule) were obtained from M/s Akay Natural Ingredients (Cochin, India) in tightly sealed HDPE bottles along with a detailed certificate of analysis. BCO-5 is a patented cold-pressed black cumin oil extract containing 5% (w/w) of thymoquinone and a unique composition of thymoquinone to carvacrol in the ratio 10:1. The study was designed as a randomized double-blind placebo-controlled trial. The consort flow diagram and the cohort study design are shown in Figure 1. Healthy participants with non-restorative sleep patterns were recruited from the database of the trial coordinator organization. The study was conducted at the BGS Global Institute of Medical Sciences, Bangalore, India, under the guidance of qualified and trained medical practitioners, following the Declaration of Helsinki and was in accordance with the clinical research guidelines of the Government of India. This study was registered in the Clinical Trial Registry of India (CTRI/2021/05/033761, dated 24/05/2021).

### 2.3 Sample size, randomization, and intervention

*A priori* power calculation analysis performed using G* power revealed that 70 participants would be required, with an estimated non-compliance or dropout rate of 20%, yielding 80% power and 5% significance (33). A total of 125 participants including male and female (18–65 years old) were screened for eligibility according to the eligibility criteria and medical history (Visit I). Seventy subjects (*n* = 70; 52 males and 18 females), characterized with nonrestorative sleep and satisfied with inclusion/exclusion criteria, were enrolled for the study. Written informed consent were obtained from selected participants and were randomized, as per permuted-block randomization technique (block size = 4),^1^ into either BCO-5 or placebo (*n* = 35/group). The details of the inclusion and exclusion criteria are presented in Table 1. A single dose (200 mg/day) of the supplement was advised to be consumed 30 min before bedtime at night, daily. The study duration was fourteen days, in which the initial seven days were used for the evaluation of baseline demography, anthropometry and vitals data and the remaining seven days were treated with either placebo or BCO-5. Duration of the study was decided on the basis of previous polysomnography (PSG) report on the improved sleep efficacy of BCO-5 when supplemented for seven days (30). Prior to the commencement of the study, all participants were provided with sleep hygiene guidelines, which encompassed practices such as exercise, stress management, reducing noise, maintaining consistent sleep timing, internet usage in the bedtime and refraining from the consumption of caffeine, nicotine, alcohol, and daytime napping.

**Table 1:** Inclusion and exclusion criteria.

Inclusion criteria Healthy males and females aged 18–65 years (both inclusive). Participants having RSQ-W scores <50. Participants who have not been taking hypnotic medicines for the last month. No prior psychiatric conditions. Participants who were ready to abstain from alcohol consumption, smoking and caffeinated beverages. Participants who can adhere to a routine diet and exercise regimen throughout the study. Participants who can understand the study procedure and provide signed informed consent to participate in the study.
Exclusion criteria Subjects suffering from any chronic health conditions like diabetes, hypertension, chronic renal failure, heart, thyroid, and liver disease and requiring medical treatment. Participants with prior history of melatonin consumption or any other sleep-enhancing nutritional supplements within the last six months. Participants with hepatic or renal impairment. Participants with a history of drug addiction/alcohol abuse/chain smokers. Individuals who are currently using or have a history of using medication, including prescription medication, herbal treatments, and over-the-counter medication, such as anxiolytics, central nervous system active drugs, hypnotics, narcotic analgesics, anti-inflammatory agents, antidepressants, proton pump inhibitors, antacids, beta-blockers, anticonvulsants, St John’s Wort, sedating H1 antihistamines, Kava-kava, systemic steroids, *Ginkgo biloba*, respiratory and prescription stimulants, decongestants, prescription diet aids, and antipsychotics, were excluded from the study. Patients with other serious concurrent illness or malignancy. Subjects with immunodeficiency diseases such as HIV or Hepatitis B positive/any other immuno-compromised state. Participants allergic to black seed or similar herbals. Pregnant and lactating women. History of clinically significant illness or any other medical disorder that may interfere with subject treatment, assessment, or compliance with the protocol. Currently participating or having participated in another clinical trial during the last one month prior to the beginning of this study.

### 2.4 Allocation concealment

The investigators were blinded to the intervention via specific codes. The treatment allocation and blinding details were maintained by an independent statistician until the completion of the clinical trial. Regular assessments were carried out during the study to ensure the effectiveness of double-blinding and to identify any instances where participants or investigators became aware of the assigned treatments.

### 2.5 Actigraphy measurements

Actigraphy is a validated method employed to measure average motor activities, and hence the sleep parameters over a period of days to weeks. An Actiwatch measures activity using a piezoelectric accelerometer set to record the intensity or duration of movement in all directions. The recorded data were analyzed using the software obtained along with the watch and manually scored by an experienced actigraphy technician. The software used the proportional integration method and a scoring algorithm validated against polysomnography (PSG) (34). The software directly provides computations for sleep efficiency, sleep onset latency, total sleep time, and wake-after-sleep-onset (WASO).

In the typical protocol, Actiwatch was provided to all participants during visit II (Day 1) and the method of use and its importance were explained. The participants were instructed to always wear the Actiwatch on the non-dominant wrist, except for bathing and to push the event marker button on the device during the bed and wake times. Fourteen-day consecutive actigraphy data were recorded. All participants were asked to report on day 8 and actigraphy recordings were noted. They were randomized into BCO-5 and Placebo groups and provided with the respective softgels capsules sealed in an air-tight container. Actigraphy procedure was then repeated for another 7 days, until Day 14 (Figure 1). Simultaneously, participants were advised to complete a sleep log during the study period and write about their feelings after consuming the medication.

### 2.6 Restorative sleep questionnaire (RSQ-W)

The Restorative Sleep Questionnaire (RSQ) is a validated 9-item questionnaire that assesses restorative sleep by asking respondents to rate on a 5-point scale about their feelings of tiredness, mood, and energy. Some items were reverse-scored, and the total score was based on the average score of nine items and rescaled to 0–100 using a typical transformation (35).


$$RSQ:W\;\mathrm{Total}\;\mathrm{Score}=\left(\begin{array}{l} RSQ:W\;\mathrm{average}\;\mathrm{score}\;\\ \mathrm{across}\;\mathrm{completed}\;\mathrm{items}:1 \end{array}\right)\times 25$$

### 2.7 Statistical analysis

Statistical analyses were performed using the IBM SPSS version 28 software. The mean and standard deviation for continuous variables and percentages for categorical variables were reported accordingly. Intra-group comparisons were performed using a paired sample *t*-test to analyze the biochemical parameters. Inter-group comparisons (independent *t*-test) were performed to analyze demographic variables across the two treatment groups for continuous variables. The significance of actigraphy and the RSQ-W was checked using a 2 × 2 repeated-measures ANOVA. ‘*P*’ values <0.05 were considered statistically significant (36).

## 3 Results

### 3.1 Participants characteristics

Participants (*n* = 70) having RSQ-W scores <50 were accrued based on the inclusion/exclusion criteria. At the end of the study, a total of seven participants were excluded. Four participants from the placebo group and three from the BCO-5 group were eliminated due to noncompliance, relocation, or changes in lifestyle patterns. Participants’ characteristics and demographics are presented in Table 2. There was no statistically significant difference (*p* > 0.05) between the placebo and BCO-5 groups at baseline.

**Table 2:** Vital signs, demographic, and anthropometric parameters of volunteers at baseline and at the end of study (EOS).

Parameters	Groups	Baseline	EOS
Age (yr.)	Placebo	37 ± 11.00	
	BCO-5	39 ± 10.00	
BMI (Kg/m^2^)	Placebo	25.8 ± 3.1	25.8 ± 3.1
	BCO-5	25.0 ± 1.5	25.1 ± 2.0
Systolic BP (mmHg)	Placebo	122 ± 6	121 ± 6
	BCO-5	122 ± 4	122 ± 5
Diastolic BP (mmHg)	Placebo	85 ± 4	84 ± 4
	BCO-5	85 ± 6	84 ± 4
Pulse (bpm)	Placebo	75 ± 4	76 ± 4
	BCO-5	75 ± 4	76 ± 3

BMI, body mass index; BP, blood pressure; values are expressed as mean ± SD. A significance level *p* < 0.05 is considered as statistically differ.

### 3.2 Actigraphy analysis

**Figure 2:**
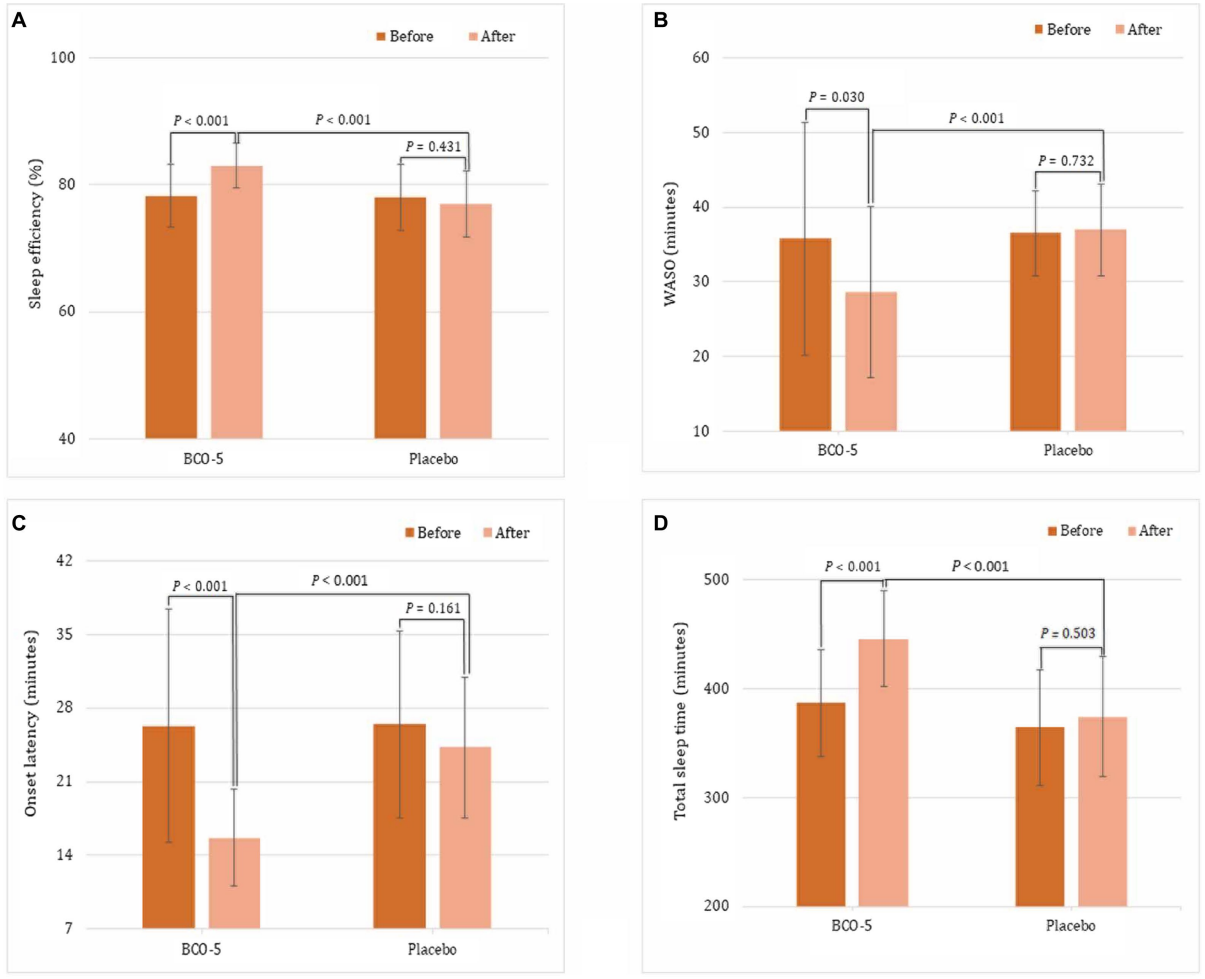
Average difference between sleep measures derived by wrist actigraphy. **(A)** sleep effiency, **(B)** WASO, **(C)** sleep onset latency, **(D)** total sleep time. Values are expressed as Mean ± SD. A ‘*P*’ value less than 0.05 (*p* < 0.05) is considered as statistically significant.

Various sleep parameters, including total sleep time, onset latency, sleep efficiency, and WASO were measured using actigraphy and compared by both intra and inter group analysis; the results are summarized in Supplementary Table S1 and Figure 2. Intra-group (baseline vs. end of study) comparison showed no significant changes in sleep efficiency (*p* = 0.431), WASO (*p* = 0.732), sleep onset latency (*p* = 0.161), or total sleep time (*p* = 0.503) in the placebo group (Supplementary Table S1). But, BCO-5 group showed significant changes (*p* < 0.001). The relative changes in BCO-5 with respect to the baseline were: sleep efficiency increased by 6.1% (*p* < 0.001); total sleep time increased by 15.2% (*p* < 0.001); sleep onset latency decreased by 40.4% (*p* < 0.001) and WASO decreased by 19.8% (*p* = 0.03). Further statistical analysis of the changes in the mean outcome measures observed in BCO-5 and placebo also revealed a significant effect (*p* < 0.001).

The baseline actigraphy measures were not significantly different (*p* > 0.05) between BCO-5 and the placebo. At the end of the study, BCO-5 exhibited a significant increase (*p* < 0.001) in sleep efficiency (7.8%) and total sleep time (19.1%) compared to placebo. Sleep onset latency and WASO on the other hand showed a significant decrease (35.4 and 22.5% respectively; *p* < 0.001) (Supplementary Table S1; Figure 2).

### 3.3 Effect on RSQ-W score

**Figure 3:**
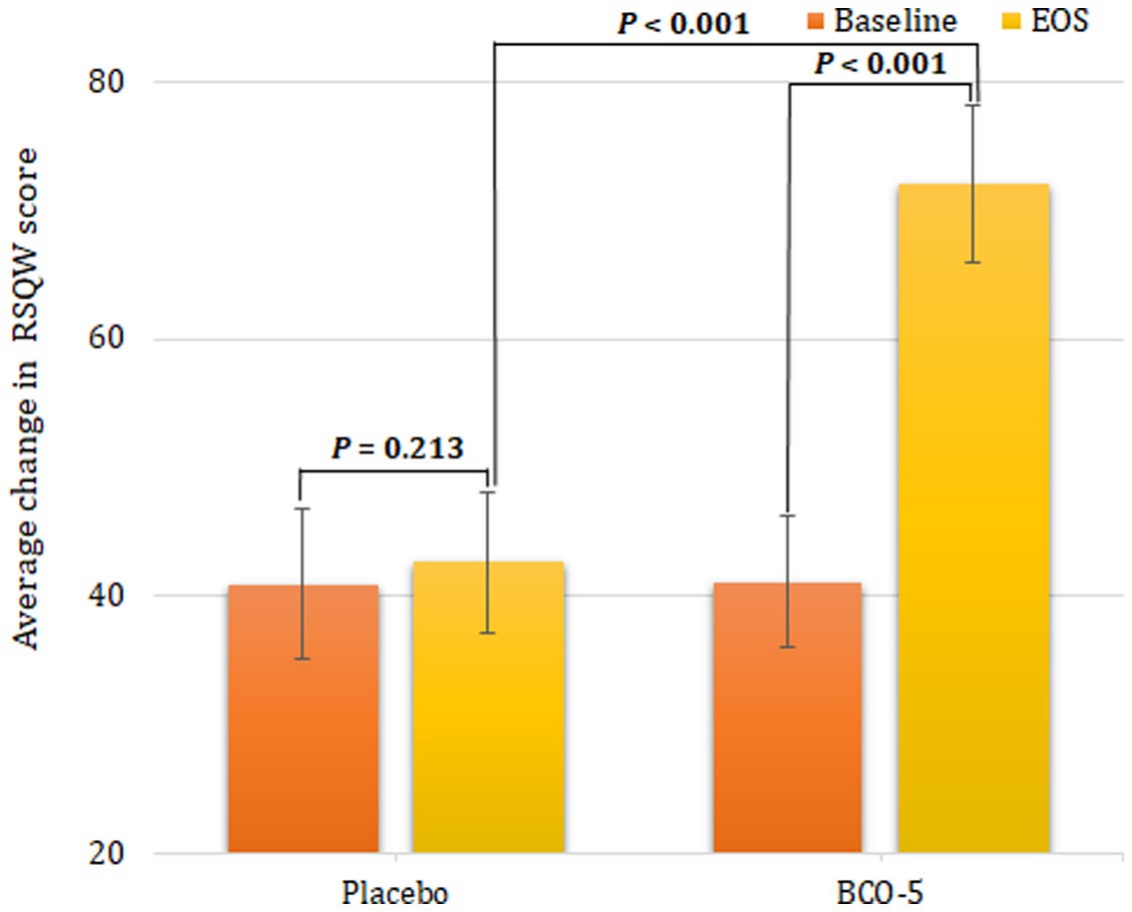
Outcome of the RSQ-W questionnaire at baseline and at the end of study. Values are expressed as Mean ± SD. A ‘*P*’ value less than 0.05 (*p* < 0.05) is considered as statistically significant.

Non-restorative sleep was assessed using the validated RSQ-W questionnaire; the results are shown in Supplementary Table S2 and Figure 3. The intra-group comparison (baseline vs. end of study) did not show a significant difference in the placebo (4.3%; *p* = 0.213), whereas the BCO-5 exhibited a significant increase (75.3%; *p* < 0.001). The mean difference in RSQ-W outcome score for BCO-5 was found to be significantly different (*p* < 0.001) with respect to placebo.

The inter-group comparison (BCO-5 vs. placebo) at the end of the study showed a significant increase in RSQ-W score (68.9%; *p* < 0.001).

### 3.4 Effect of BCO-5 on vital parameters

There was no statistically significant difference (*p* > 0.05) in the vital signs between the placebo and BCO-5 groups either at baseline or at the end of the study (Table 2).

### 3.5 Adverse effects

No adverse effects were reported by the participants for the placebo and BCO-5, except for the after-taste of black cumin extract, which was reported by two participants.

## 4 Discussion

The present randomized, double-blind, placebo-controlled clinical trial was conducted to investigate the short-term effects of BCO-5 on participants reported with non-restorative sleep problems when supplemented at 200 mg/day after dinner for seven days. It has been reported that, sleep disorders and distinct sleep patterns in conditions such as nonrestorative sleep can be identified and quantified using subjective (questionnaires) and objective measures (instruments) (35). Considering this, our present study employed actigraphy as objective measure along with the restorative sleep questionnaire (RSQ-W) as a subjective measure.

All participants were characterized by typical nonrestorative sleep issues, as evident from the weekly version of the restorative sleep questionnaire (RSQ-W). RSQ was developed to validate nonrestorative sleep, which helps assessment on how restored an individual would feel after waking up (37, 38). It has good psychometric properties and can distinguish healthy controls from people with primary insomnia, and people with isolated non-restorative sleep complaints. The RSQ-W score observed in the present study shows significant restoration of sleep and improved quality of life indicating the primary efficacy of treatment with a single dose (200 mg × 1/day) of BCO-5 approximately half an hour before bedtime for 7 days. These findings were further supported by actigraphy data, which revealed remarkable enhancement in sleep quality as evident from various sleep parameters, including sleep onset latency, WASO and total sleep time.

To better understand the significance of our protocol and results, we reviewed previous actigraphy studies on certain herbal extracts currently using for the treatment of sleep disorders. Supplementation of passion flower extract at 60 mg/day for 14 days was reported to improve sleep efficiency and WASO (39). Tart cherry juice consumption for 7 days has shown to significantly increase total sleep time and sleep efficiency (40). In another study, significant improvement in sleep efficiency was reported when a rice bran extract was supplemented for 14 days at 1000 mg/day (41). These studies justify our protocol involving 7 days’ supplementation to investigate the influence of BCO-5 on sleep.

Valerian is one of the herbal extracts widely using as herbal supplement for sleep issues, though its clinical studies at various dosage ranging from 150 to 600 mg/day have provided mixed results. While the subjective measurements reported positive effect, the objective measures such as actigraphy and polysomnography failed to show efficacy (42). Another commercially available extract to treat insomnia is saffron. Most of its studies are based on the subjective measurements with mixed results. Kell et al. reported no effect on sleep quality when 128 subjects were treated with a standardized saffron extract (affron®) for four weeks at 22 and 28 mg/day though there was a decrease in negative mood and symptoms related to stress/anxiety (43). Another standardized saffron extract (Saffr’active®), resulted in significant increase in ‘time in bed’ when treated with 15.5 mg/day for six weeks; none of the other parameters showed improvement (44). Ashwagandha is yet another widely using herbal extract for better sleep. Deshpande et al. reported significant improvement in various actigraphy parameters when treated with a standardized ashwagandha leaf extract (Shoden®) for 6 weeks (45). In another study, standardized ashwagandha root extract (KSM66®) supplementation at 600 mg per day for 70 days reported to improve sleep (46). Thus, our results on BCO-5 were found to be superior to many other herbal extracts presented in the last decade, especially in the last 3 years, when considering the low dose of 200 mg/day, short-term duration of the study protocol (7 days), sample size (*n* = 70), randomized controlled design with both subjective and objective measurements of sleep and finally the statistically significant correlation observed between both the measurements.

Actigraphy results of the present study were also in agreement with a previous single-arm, open-label pilot study where BCO-5 improved both Non-Rapid Eye Movement (NREM) and REM sleep by 82.5 and 29.4%, respectively. A significant reduction (*p* < 0.05) in anxiety, stress and cortisol was also reported in the study (30). Increased cortisol level was generally correlated with reduced NREM and REM stages. In another recent randomized controlled study employing Pittsburgh sleep quality questionnaire (PSQI), more than 70% of participants treated with BCO-5 at 200 mg/day were satisfied with their sleep improvement in 7 days (47).

The sleep effect of BCO-5 may be attributed to its essential oil content. Certain essential oils such as lavender, chamomile and orange peel have already been reported to possess sleep promoting effect, when administered orally (48–50), though their major practice is in aromatherapy for sleep. Terpenes and terpenoids are the bioactive principles in essential oils. GC–MS/MS analysis showed that BCO-5 contains carvacrol, α-thujene and *p-*cymene as the major terpenes and terpenoids in addition to thymoquinone, in a unique ratio of 10:1:1:2 (v/v).

Recently we had reported the influence of BCO-5 on the hypothalamus-pituitary–adrenal (HPA)-axis to act as a dual orexin receptor antagonist (DORA) and hence to modulate cortisol/melatonin levels and thereby to balance stress/sleep and sleep/wake cycle (47). Inhibition of orexin activation can reduce cortisol and promote sleep signals by enhancing melatonin. Orexin agonism on the other hand promote wakefulness (51). Serotonergic and GABAergic effect of black cumin and BCO-5 have been reported previously which also play an important role in stress and sleep modulation (52–56).

The fact that BCO-5 did not cause any significant side effects or adverse events further supports its safety. In an animal study (acute and sub chronic toxicity), as per the Organization for Economic Cooperation and Development (OECD) guidelines, it was reported that BCO-5 is safe at 94 mg/kg body weight, indicating suitability for human consumption (32). Recently, a randomized, double-blind, placebo-controlled trial also evaluated the safety of BCO-5 at 200 mg/adult/day for 90 days in healthy volunteers and showed no adverse events or deviations in biochemical and hematological parameters (31). However, it was effective in significantly reducing (*p* < 0.05) total cholesterol, low-density lipoproteins, very-low-density lipoproteins, and triglycerides (*p* < 0.05).

The measurement of sleep within a short time of one week using both subjective and objective measures following a randomized, double-blind, placebo-controlled design exist as the major strength of the present study. However, lack of measurements of cortisol and melatonin, lack of measurement of the systemic absorption, distribution and elimination of the suspected bioactive molecules in BCO-5 and the absence of inclusion of a washout period in the study protocol may be considered as limitations of the present study.

## 5 Conclusion

Sleep disorders have become a common global problem, owing to increased stress and hormonal imbalance. One of the most widely observed sleep disorder is non-restorative or non-refreshed insomnia. The present randomized, double-blind, placebo-controlled study illustrated the beneficial effects of BCO-5, a unique composition of black cumin oil extract, in alleviating nonrestorative sleep in just a week time when supplemented at 200 mg/day. Actigraphy study could establish the improvement in sleep efficiency, sleep onset latency, total sleep time, and WASO without adverse side effects. However, future studies on various population types are warranted to determine their full therapeutic potential for the management of sleep issues and related disorders.

## Data availability statement

The original contributions presented in the study are included in the article/Supplementary material, further inquiries can be directed to the corresponding author.

## Ethics statement

The studies involving humans were approved by Balagangadharanatha Swami Global Institute of Medical Sciences, dated 10th May, 2021. The studies were conducted in accordance with the local legislation and institutional requirements. The participants provided their written informed consent to participate in this study.

## Author contributions

IK and PB: conceptualization. MM: project administration, investigation, and data curation. PP: writing—original draft preparation. SS, MCM, and IK: writing—review and editing. All authors have read and agreed to the published version of the manuscript.

## Footnotes

^1^http://www.Randomisation.com
