# Corrigendum: Exploring the short-term influence of a proprietary oil extract of black cumin (*Nigella sativa*) on non-restorative sleep: a randomized, double-blinded, placebo-controlled actigraphy study

**DOI:** 10.3389/fnut.2024.1378259

**Published:** 2024-02-13

**Authors:** M. E. Mohan, Mohind C. Mohan, Prathibha Prabhakaran, S. Syam Das, I. M. Krishnakumar, P. S. Baby Chakrapani

**Affiliations:** ^1^Department of General Medicine, BGS Global Institute of Medical Sciences, Kengeri, India; ^2^Centre for Neuroscience, Cochin University of Science and Technology, Cochin, Kerala, India; ^3^Department of Biotechnology, Cochin University of Science and Technology, Cochin, Kerala, India; ^4^R&D Centre, Akay Natural Ingredients, Cochin, Kerala, India; ^5^Centre of Excellence in Neurodegeneration and Brain Health, Cochin, Kerala, India

**Keywords:** actigraphy, black cumin, insomnia, non-restorative sleep, non-refreshing sleep, *Nigella sativa*, thymoquinone

In the published article, there was an error in Figure 2A as published. In Figure 2A the *P* value of the placebo group is wrongly written as *P* < *0.001*. The corrected Figure 2 and its caption appear below.

**Figure 2:**
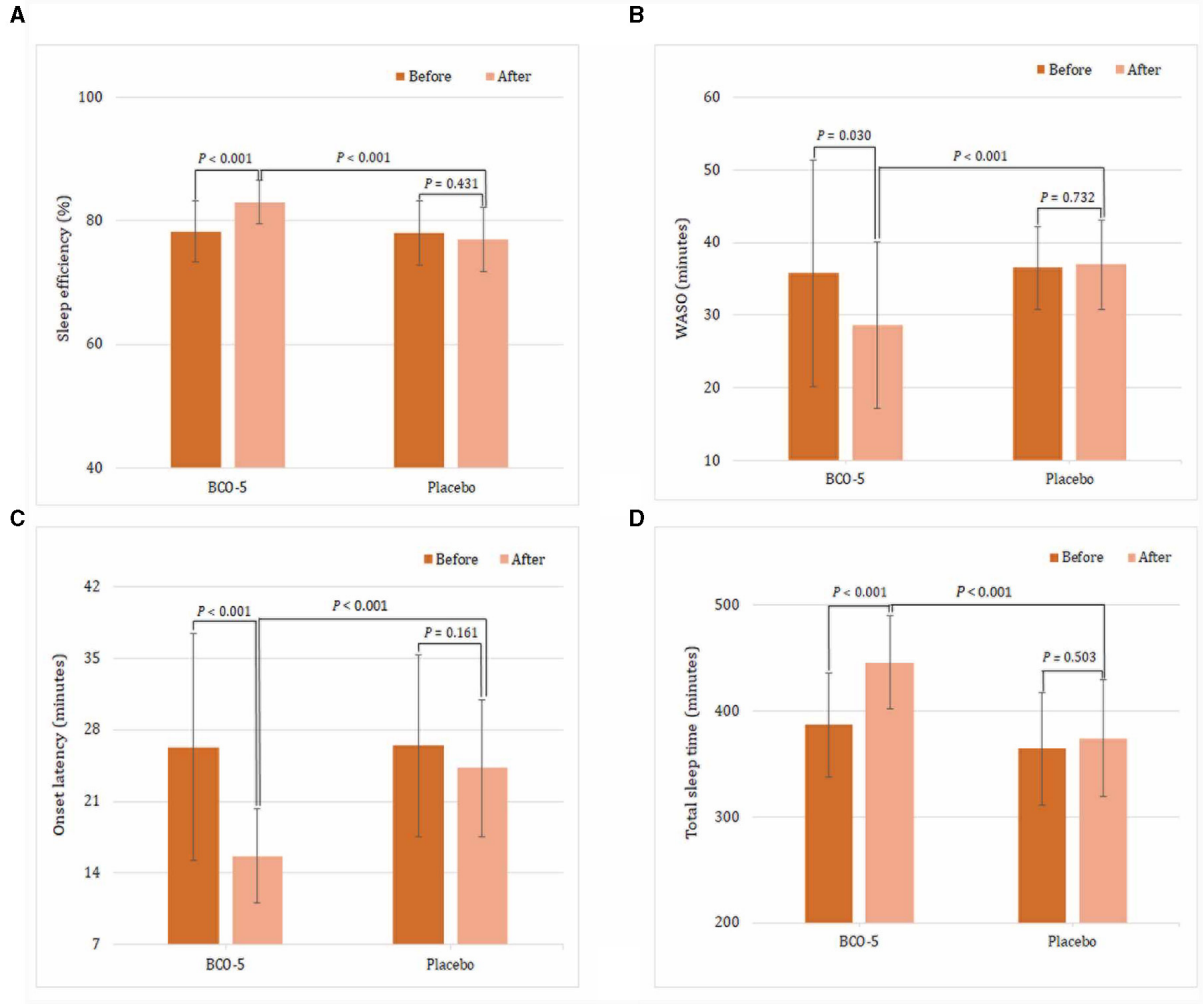
Average difference between sleep measures derived by wrist actigraphy. **(A)** sleep efficiency, **(B)** WASO, **(C)** Sleep onset latency, **(D)** total sleep time. Values are expressed as Mean ± SD. A “*P*” value less than 0.05 (*P* < 0.05) is considered as statistically significant.

The authors apologize for this error and state that this does not change the scientific conclusions of the article in any way. The original article has been updated.

