# Supplementary material for: Exploring the short-term influence of a proprietary oil extract of black cumin (Nigella sativa) on non-restorative sleep: a randomized, double-blinded, placebo-controlled actigraphy study
Source: Front Nutr. 2024 Jan 15;10:1200118. doi: 10.3389/fnut.2023.1200118 (PMC10822901; doi:10.3389/fnut.2023.1200118)
Supplement: Supplementary file 1 [file Table_1.DOCX]

Supplementary Material

Exploring the short-term influence of a proprietary oil extract of Black cumin (*Nigella sativa*) on non-restorative sleep: A randomized, double-blind, placebo-controlled actigraphy study

Mohan ME^1^, Mohind C Mohan^2,3^, Prathibha Prabhakaran^4^, Syam Das S^4^, Krishnakumar IM^4^, Baby Chakrapani PS^2,3*^

*** Correspondence:** Baby Chakrapani PS, Ph.D.Email: [chakrapani@cusat.ac.in](mailto:chakrapani@cusat.ac.in), [bcps80@gmail.com](mailto:bcps80@gmail.com)

**Table 1S.** Actigraphy measurements upon intra (Baseline vs End of the study) and inter group (Placebo vs BCO)

| **2Parameter** | **Groups** | **Baseline**  **(Day 7)** | **EOS**  **(Day 14)** | ***P* -value**  **(intra-group)** | **% Change** | ***P-* value (inter-group)** | |
| --- | --- | --- | --- | --- | --- | --- | --- |
|  |  |  |  |  |  | ***Baseline*** | ***EOS*** |
| **Sleep efficiency (%)** | **Placebo** | 78.1 ± 5.2 | 77.0 ± 5.2 | *P* = 0.431 | 6.09 | *P* = 0.482 | *P* < 0.001 |
|  | **BCO-5** | 78.3 ± 4.9 | 83.0 ± 3.6 | *P* < 0.001 |  |  |  |
| **Sleep onset latency (min)** | **Placebo** | 26.5 ± 8.9 | 24.3 ± 6.7 | *P* = 0.161 | 40.4 | *P* = 0.653 | *P* < 0.001 |
|  | **BCO-5** | 26.3 ± 11.1 | 15.7 ± 4.6 | *P* < 0.001 |  |  |  |
| **Total sleep time (min)** | **Placebo** | 364.3 ± 53.0 | 374.4 ± 54.8 | *P* = 0.503 | 15.21 | *P* = 0.069 | *P* < 0.001 |
|  | **BCO-5** | 387.0 ± 49.0 | 445.9 ± 44.1 | *P* < 0.001 |  |  |  |
| **WASO (min)** | **Placebo** | 36.5 ± 5.7 | 37.0 ± 6.2 | *P* = 0.732 | 19.84 | *P* = 0.521 | *P* < 0.001 |
|  | **BCO-5** | 35.8 ± 15.6 | 28.7 ± 11.4 | *P* = 0.030 |  |  |  |

Values are expressed as mean ± SD. A ‘*P*’ value less than 0.05 (*P* < 0.05) was considered statistically significant.

**Table 2S.** Intra and inter-group comparison of RSQ-W scores

| **Groups** | **Baseline** | **EOS** | **Intra-group *P* value** | **Inter-group comparison**  ***P* value** | | **% Change (Intra-group)** |
| --- | --- | --- | --- | --- | --- | --- |
|  |  |  |  | **Baseline** | **EOS** |  |
| **Placebo** | 40.9 ± 5.9 | 42.7 ± 5.5 | *P* = 0.213 | *P* = 0.925 | *P* < 0.001 | 4.23 |
| **BCO-5** | 41.1 ± 5.1 | 72.1 ± 6.1 | *P* < 0.001 |  |  | 75.26 |

Values are expressed as mean ± SD. A ‘*P*’ value less than 0.05 (*P* < 0.05) was considered statistically significant.
